# Supplementary material for: LONP1 and mtHSP70 cooperate to promote mitochondrial protein folding
Source: Nat Commun. 2021 Jan 11;12:265. doi: 10.1038/s41467-020-20597-z (PMC7801493; doi:10.1038/s41467-020-20597-z)
Supplement: Supplementary file 1 — Supplementary Information [file 41467_2020_20597_MOESM1_ESM.pdf]

Supplementary Figure 1

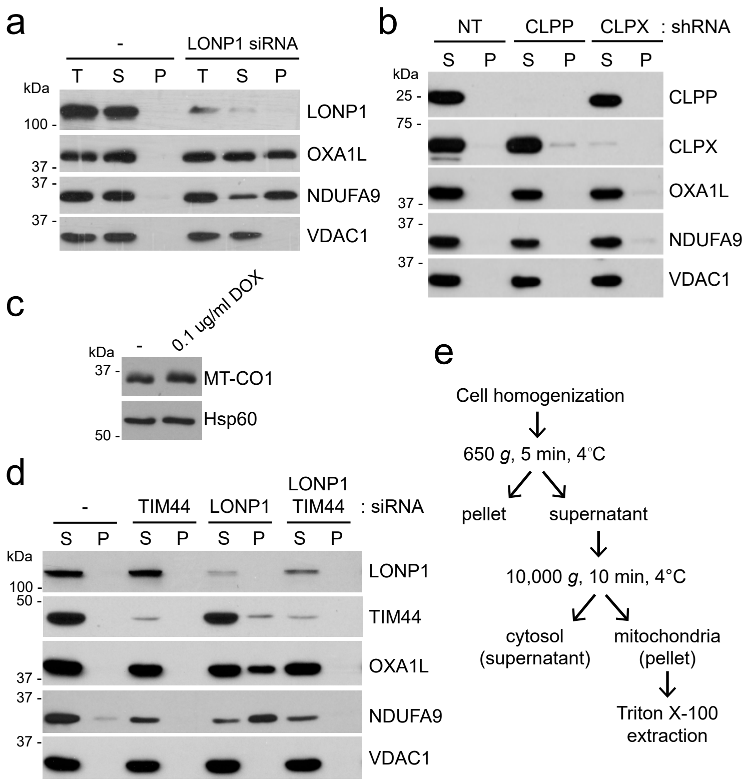

Supplementary Figure 1. Protein insolubility in mitochondrial protease knockdown cells and mitochondrial isolation

(a, b) Mitochondrial protein solubility in LONP1 and CLPXP knockdown cells. Triton X-100 extraction and Western blotting were used to examine protein solubility in LONP1 knockdown (a), CLPP or CLPX knockdown (b) 143B cells. T, total; S, supernatant; P, pellet. NT: non-target. (c) Mitochondrial translation in the presence of DOX. 143B cells were treated with 0.1 µg/ml DOX and the expression of mitochondrial DNA-encoded MT-CO1 was analyzed by Western blotting. (d) Effect of TIM44 knockdown on mitochondrial protein solubility in LONP1 knockdown cells. (e) Workflow of mitochondrial isolation and Triton X-100 extraction.

Supplementary Figure 2

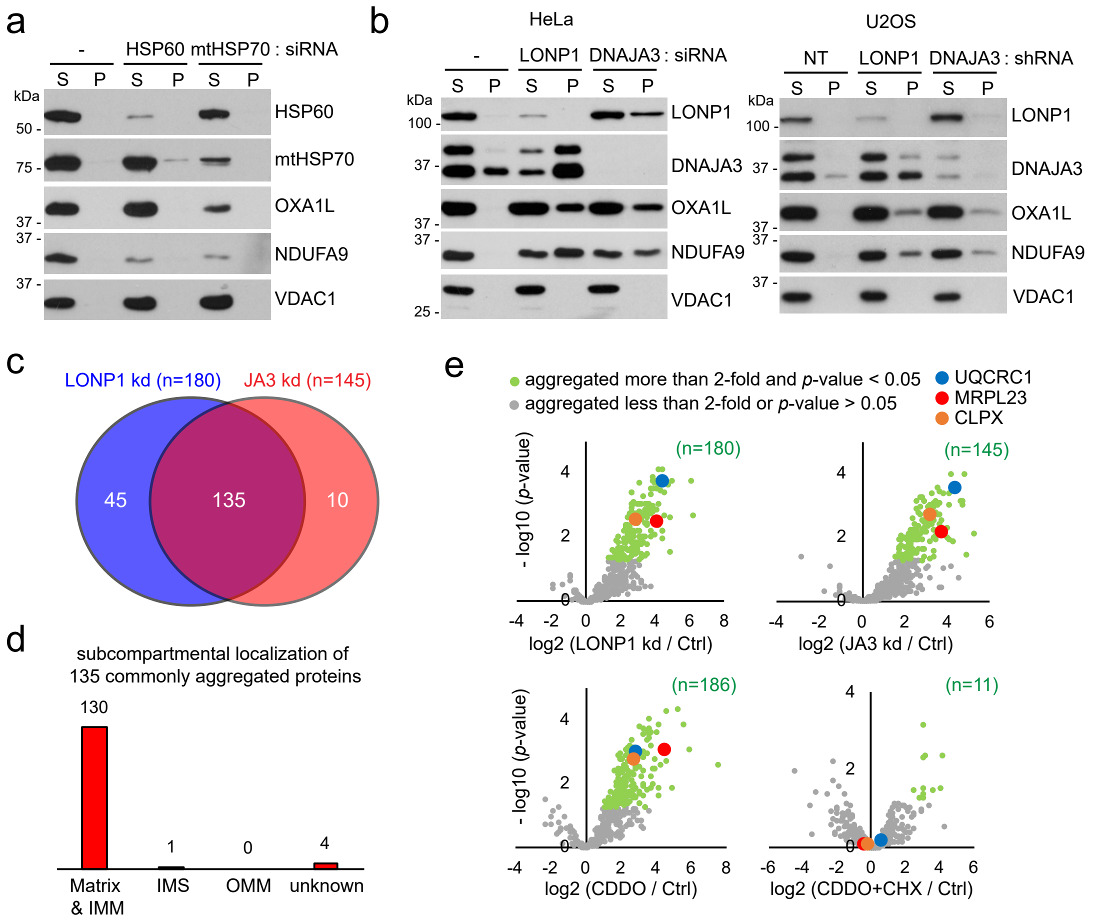

Supplementary Figure 2. Aggregation profile of mitochondrial proteins in LONP1 knockdown, DNAJA3 knockdown, and CDDO-treated cells

(a) Mitochondrial protein solubility in HSP60 and mtHSP70 knockdown 143B cells. (b) Comparison of mitochondrial protein insolubility in DNAJA3 versus LONP1 knockdown cells. Cell lines are indicated. NT: non-target. (c) Venn diagram analysis of aggregated proteins in LONP1 and DNAJA3 knockdown mitochondria. Proteins depicted were at least 2-fold over control in mitochondrial pellets, with a p-value < 0.05. Statistical analysis of proteins enriched in mitochondrial aggregates was performed using the LIMMA moderated two-tailed t-test. (d) Plot of subcompartmental localization of the 135 proteins aggregated in both DNAJA3 and LONP1 knockdown mitochondria. (e) Volcano plot representations of 455 mitochondrial proteins detected in the insoluble fraction of LONP1 knockdown, DNAJA3 knockdown, CDDO-treated, and CDDO/CHX-treated mitochondria. Three proteins, including CLPX, are highlighted.

Supplementary Figure 3

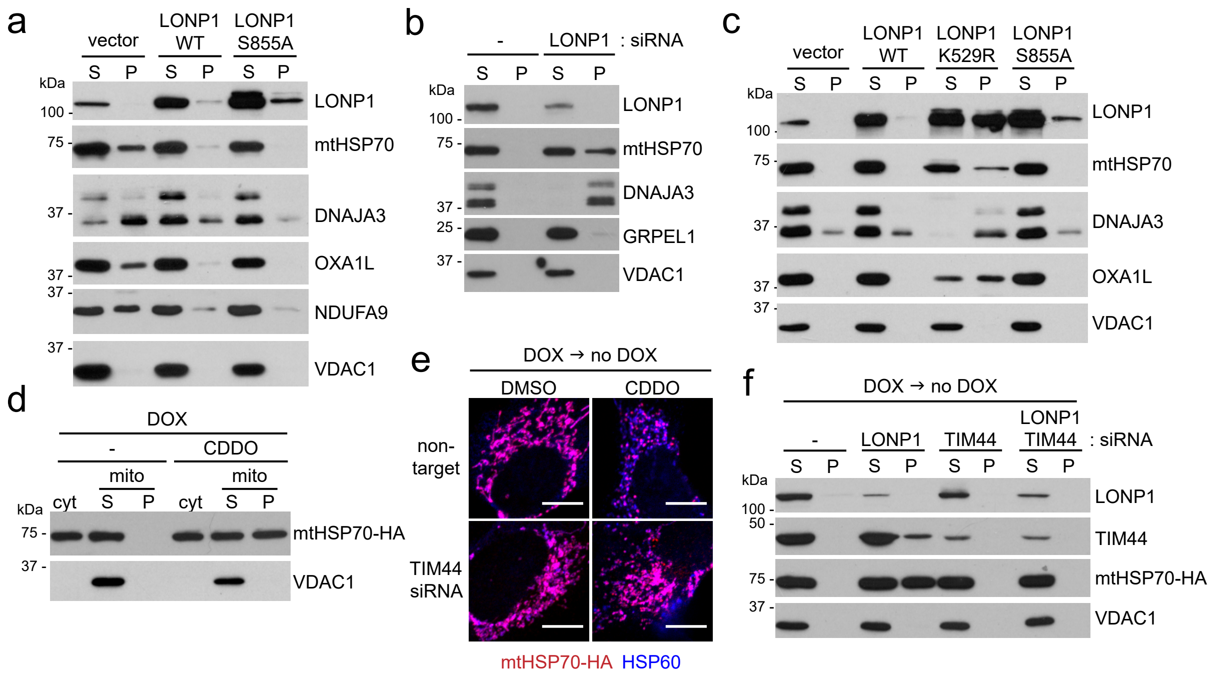

Supplementary Figure 3. mtHSP70 aggregation with LONP1 inhibition

(a) Rescue of protein insolubility by protease-deficient LONP1. 143B cells expressing LONP1^{WT} and LONP1^{S855A} were treated with 2 μM CDDO for 12 hr and analyzed by Triton X-100 extraction and Western blotting. (b) Solubility of mtHSP70 chaperone components in LONP1 knockdown 143B cells. (c) Effect of LONP1 variants on mitochondrial protein solubility. 143B cells expressing LONP1^{WT}, ATPase-deficient LONP1^{K529R}, or protease-deficient LONP1^{S855A} were analyzed by Triton X-100 extraction and Western blotting. (d) Solubility of mtHSP70-HA upon LONP1 inhibition. mtHSP70-HA in 143B cells was induced by DOX for 16 hr in the absence or presence of 2 μM CDDO. Isolated mitochondria were extracted with Triton X-100 and analyzed by Western blotting. cyt (cytosol), mito (mitochondria). (e) Rescue of CDDO-induced mtHSP70 insolubility by TIM44 knockdown. mtHSP70-HA expression was induced in 143B cells by a pulse of DOX. After removal of DOX, the cells were treated with 2 μM CDDO for 6 hr and analyzed by immunostaining against HA and HSP60. Scale bar, 10 μm. (f) Rescue of CDDO-induced mtHSP70 insolubility by TIM44 knockdown. mtHSP70-HA expression was induced by DOX for 24 hr. After DOX removal, the cells were transfected with the indicated siRNAs and cultured for 3 days before Triton X-100 extraction and Western blot analysis.

Supplementary Figure 4

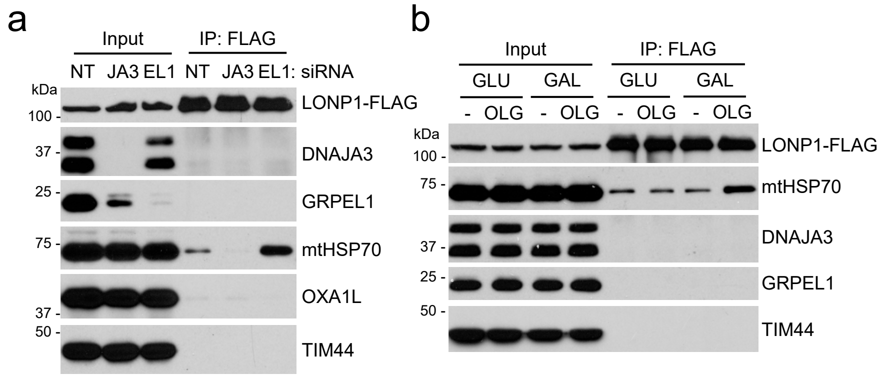

Supplementary Figure 4. LONP1 co-immunoprecipation with mtHSP70

(a) Analysis of LONP1-FLAG interactions in response to DNAJA3 and GRPEL1 knockdown. LONP1-FLAG was constitutively expressed in the indicated 143B cells, immunoprecipitated, and analyzed by Western blotting. NT, non-target; JA3, DNAJA3; EL1, GRPEL1. (b) Effect of galactose culture and oligomycin treatment on the LONP1-mtHSP70 interaction. LONP1-FLAG immunoprecipitates were analyzed for the indicated proteins.

Supplementary Figure 5

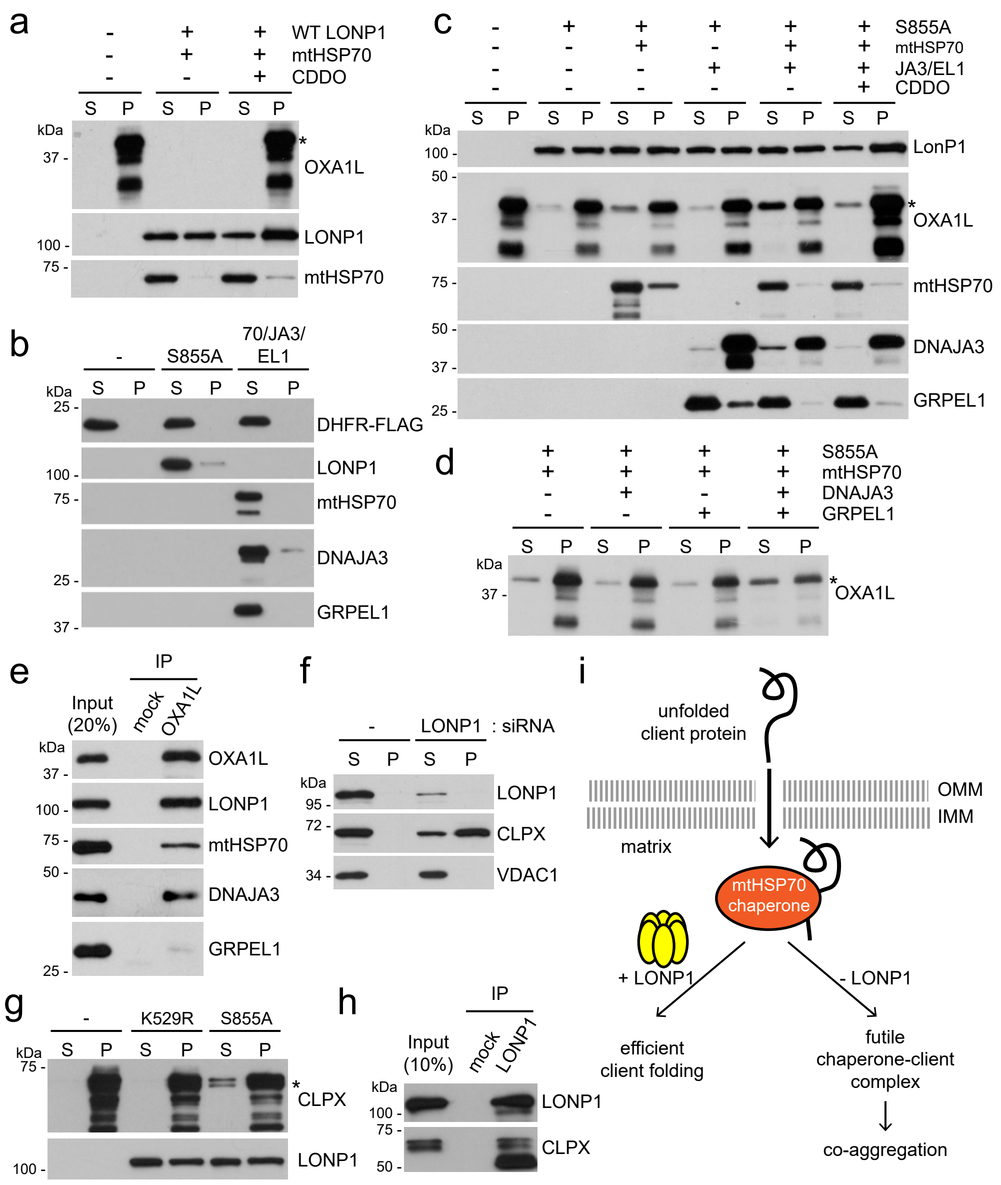

Supplementary Figure 5. ***In vitro*** **reconstitution of OXA1L and CLPX solubilization**

(a) Effect of recombinant WT LONP1 (1 μM) and mtHSP70 (4 μM) on OXA1L *in vitro* translation reaction. For CDDO treatment, LONP1 was pre-incubated with 40 μM CDDO for 1 hr at 37°C and then added to the i*n vitro* translation reaction. An asterisk (*) indicates full-length OXA1L. (b) DHFR-FLAG as a control protein for the *in vitro* reaction. Reactions contained recombinant LONP1^{S855A} (2 μM) or mtHSP70 chaperone components (4 μM mtHSP70, 1 μM DNAJA3, 1 μM GRPEL1) as indicated. (c) Effect of protease-deficient LONP1^{S855A} and mtHSP70 chaperone components (4 μM mtHSP70, 1 μM DNAJA3, 1 μM GRPEL1) on OXA1L solubility. For CDDO treatment, LONP1 was pre-incubated with 40 μM CDDO for 1 hr at 37°C and then added to *in vitro* translation reaction. (d) Individual effect of the mtHSP70 co-chaperones DNAJA3 (1 μM) and GRPEL1 (1 μM) on the *in vitro* reaction. (e) Analysis of the interactions of solubilized OXA1L. OXA1L was immunoprecipitated from a reaction containing LONP1^{S855A} and mtHSP70 chaperone components, and analyzed by Western blotting. Reactions were cleared by a high speed spin prior to immunoprecipitation. (f) Solubility of CLPX in LONP1 knockdown 143B cells. (g) Effect of recombinant protease-deficient LONP1^{S855A} and ATPase-deficient LONP1^{K529R} on CLPX solubilization *in vitro*. An asterisk (*) indicates full-length CLPX. (h) Analysis of the interactions of solubilized CLPX. CLPX *in vitro* reaction containing recombinant LONP1^{S855A} (2 μM) was cleared by a high speed spin. LONP1^{S855A} was immunoprecipitated and co-immunoprecipitated CLPX was analyzed by Western blotting. (i) Model for the collaboration of LONP1 and mtHSP70 in mitochondrial protein biogenesis. Folding of the incoming mitochondrial precursor is efficient only in the presence of LONP1. In the absence of LONP1, mtHSP70 interactions with the client are futile and result in co-aggregation with the client.

Supplementary Table 1. Sequences of PCR primers used in this study

Primer	Sequence 5' to 3'
**For human cell expression**	
OXA1L-FLAG-F	CATGCGGCCGCACCATGGCGATGGGACTAATGTGC
OXA1L-FLAG-R	CATACCGGTTCACTTGTCGTCATCGTCTTTGTAGTCAGAACCAGAACCGCCGCCAAGTGTGTCGTGCCAGG
LONP1-FLAG-F	CATGCGGCCGCACCATGGCGGCGAGCACTGGCTAC
LONP1-FLAG-R	CATACCGGTTCACTTGTCGTCATCGTCTTTGTAGTCAGAACCAGAACCGCCCCGTTCCACGGCCAGCGCCT
mtHSP70-HA-F	CATGCGGCCGCACCATGATAAGTGCCAGCCGAGC
mtHSP70-HA-R	CATACCGGTTCAAGCGTAATCTGGAACATCGTATGGGTAAGAACCAGAACCGCCCTGTTTTTCCTCCTTTTGATCTTC
**For in vitro or bacterial expression**	
OXA1L-F	GTACATATGGCAGAAGTCCAGGTTCAGGC
OXA1L-R	GTAGCGGCCGCTCAGCCAAGTGTGTCGTGCCAGG
CLPX-F	GTACATATGTTTACAGAAACACCAGCATAC
CLPX-R	CATGGATCCTCATTAGCTGTTTGCAGCATCTGCTTG
DNAJA3-F	GTAGGATCCGGGTGTCAGCCTTACAGGAACAA
DNAJA3-R	GTAGCGGCCGCTCAGTTTCCAGTGGATCTTTTTCCAGAGC
**LONP1 mutagenesis**	
K529R-F	CCTGGCGTGGGTAGGACCAGCATTGCT
K529R-R	AGCAATGCTGGTCCTACCCACGCCAGG
S855A-F	CCAAGGACGGCCCAGCCGCAGGCTGCACCAT
S855A-R	ATGGTGCAGCCTGCGGCTGGGCCGTCCTTGG
R721G-F	GTGGAGAAGGTGTTAGGGAAATCGGCCTACA
R721G-R	TGTAGGCCGATTTCCCTAACACCTTCTCCAC
RNAi resistant-F	GGGACATAATCGCCTTGAACCCTCTC
RNAi resistant-R	GGTTCAAGGCGATTATGTCCCGGATGGTC
