# Supplementary material for: LONP1 and mtHSP70 cooperate to promote mitochondrial protein folding
Source: Nat Commun. 2021 Jan 11;12:265. doi: 10.1038/s41467-020-20597-z (PMC7801493; doi:10.1038/s41467-020-20597-z)
Supplement: Supplementary file 7 — Description of Additional Supplementary Files [file 41467_2020_20597_MOESM7_ESM.docx]

Description of Additional Supplementary Information

Title: Supplementary Data 1.

Description: Proteomic analysis of mitochondrial protein aggregation. Proteins detected from mass spectrometry analysis of the mitochondrial pellet fraction are listed. For the four experimental conditions, the fold enrichment and p-value for each protein are indicated. Statistical analysis was performed using the LIMMA moderated two-tailed ttest.

Title: Supplementary Data 2.

Description: Aggregated proteins found in common from mitochondria of LONP1 and DNAJA3 knockdown cells. Tabulation of the 135 proteins aggregated in the mitochondria of both LONP1 knockdown and DNAJA3 knockdown cells, including the fold enrichment, p-values, and suborganellar localization. Statistical analysis of proteins was performed using the LIMMA moderated two-tailed ttest.

Title: Supplementary Data 3.

Description: All proteins identified from samples of mitochondrial aggregates. Exported protein groups table from the PD-Byonic search result for all 15 samples analyzed by LC-MS.

Title: Supplementary Data 4.

Description: All peptides identified from samples of mitochondrial aggregates. Exported peptide groups table from the PD-Byonic search result for all 15 samples analyzed by LC-MS.
